# Transcriptome Profiling and Identification of Transcription Factors in Ramie (*Boehmeria nivea* L. Gaud) in Response to PEG Treatment, Using Illumina Paired-End Sequencing Technology

**DOI:** 10.3390/ijms16023493

**Published:** 2015-02-04

**Authors:** Xia An, Jie Chen, Jingyu Zhang, Yiwen Liao, Lunjin Dai, Bo Wang, Lijun Liu, Dingxiang Peng

**Affiliations:** Key Laboratory of Crop Ecophysiology and Farming Systems in the Middle Reaches of the Yangtze River, Ministry of Agriculture; College of Plant Science and Technology, Huazhong Agricultural University, Wuhan 430070, China; E-Mails: anxia111@126.com (X.A.); lqlcj@126.com (J.C.); 13164612991@163.com (J.Z.); lywen2009@126.com (Y.L.); dailunjin@gmail.com (L.D.); liulijun@mail.hzau.edu.cn (L.L.)

**Keywords:** ramie, PEG, paired-end sequencing, transcription factor

## Abstract

Ramie (*Boehmeria nivea* L. Gaud), commonly known as China grass, is a perennial bast fiber plant of the Urticaceae. In China, ramie farming, industry, and trade provide income for about five million people. Drought stress severely affects ramie stem growth and causes a dramatic decrease in ramie fiber production. There is a need to enhance ramie’s tolerance to drought stress. However, the drought stress regulatory mechanism in ramie remains unknown. Water stress imposed by polyethylene glycol (PEG) is a common and convenient method to evaluate plant drought tolerance. In this study, transcriptome analysis of cDNA collections from ramie subjected to PEG treatment was conducted using Illumina paired-end sequencing, which generated 170 million raw sequence reads. Between leaves and roots subjected to 24 (L2 and R2) and 72 (L3 and R3) h of PEG treatment, 16,798 genes were differentially expressed (9281 in leaves and 8627 in roots). Among these, 25 transcription factors (TFs) from the AP2 (3), MYB (6), NAC (9), zinc finger (5), and bZIP (2) families were considered to be associated with drought stress. The identified TFs could be used to further investigate drought adaptation in ramie.

## 1. Introduction

Abiotic stresses, such as drought and high salt, are becoming increasingly common because of global climate change, and severely inhibit plant growth and development [1,2]. Among all the abiotic stresses, drought has probably the most significant effect on plant distribution, growth, and productivity in natural and agricultural systems. Studies on the molecular responses to drought are vital, and will ultimately lead to enhanced stress tolerance in crops [3]. However, the responses and adaptations of plants subjected to drought conditions are complex. During the process of domestication, plants have developed numerous physiological and biochemical strategies to cope with adverse conditions [1,4]. For example, when the leaf-to-air vapor pressure or relative humidity changes, a plant’s leaves close their stomata to reduce water loss through transpiration; when suffering from progressive water loss, the leaves begin to curl to protect the photosynthetic machinery [5]. Meanwhile, the water content of cells is maintained by synthesizing and accumulating various small molecule compounds, such as soluble sugars [6] and proline [7]. Nevertheless, the production of reactive oxygen species (ROS), which are caused by severe drought stress [8], could have detrimental effects on plant growth and development [9]. To cope with oxidative damage, several enzymes are activated in plant cells, including superoxide dismutase (SOD), catalase (CAT), ascorbate peroxidase (APX), glutathione reductase (GR), and non-enzymatic antioxidants, such as ascorbic acid and glutathione [8]. Studies on the molecular mechanisms of plant responses to these stresses and damages are increasing. Recently, analyses of a large number of transcriptome sequence data sets have revealed many new stress-responsive genes in *Arabidopsis* [10] and rice [11]. However, there have been few reports on ramie [12,13].

Ramie is an important natural fiber crop, widely planted in China, India, and other Southeast Asian and Pacific Rim countries [12]. It is the second major fiber crop after cotton in China and plays an important role in Chinese economy. Ramie fibers possess high tensile strength, antibacterial properties, and good moisture absorption characteristics. In China, ramie is mainly planted by the Yangtze River and can be harvested three times per year. However, the per capita arable land area is shrinking as urbanization and industrialization accelerate. Therefore, it is a good practice to plant ramie on sloping land so that more land can be used to grow grain. However, ramie yields have shown dramatic decreases recently because of reduced rainfall in the summer or autumn. Thus, drought tolerance research in ramie (especially research into breeding drought-resistant varieties) has become particularly important. However, to date, only a few reports have focused on ramie physiological traits [12,13]. In a previous study, research into the ramie universal transcriptome has identified 24 genes that could be transcription factors (TFs), but only 12 of them have been shown to be authentically involved in drought stress responses [13].

In this study, hydroponic ramie seedlings, propagated from stem cuttings of cultivar Huazhu No. 5 and transplanted into half-strength Hoagland’s solution, were generated and subjected to simulated drought stress (PEG treatments). Four physiological traits were observed (the relative water content, RWC; the peroxidase activity, POD; the malondialdehyde content, MDA; and the proline content) from the leaves of the seedlings to distinguish the stress severity and for collecting samples for transcriptome sequencing. A total of 16,798 (9281 in leaves and 8627 in roots, respectively) unigenes were differentially expressed in the transcriptome data. Among these, 25 TFs from the AP2 (3), MYB (6), NAC (9), zinc finger (5), and bZIP (2) families were considered to be associated with drought stress because of their expression patterns (coincided with the propensities of the observed physiological traits) in leaves or roots. This study increased our understanding of the molecular responses to drought in ramie, which will improve drought resistance breeding in ramie.

## 2. Results

### 2.1. Physiological Responses of Ramie to Drought Stress

Leaves harvested at 0, 12, 24, 48, and 72 h after being treated with 15% PEG6000 (*w*/*v*) were prepared for measuring the four physiological traits (the RWC, the POD activity, the MDA content, and the proline content).

**Figure 1:**
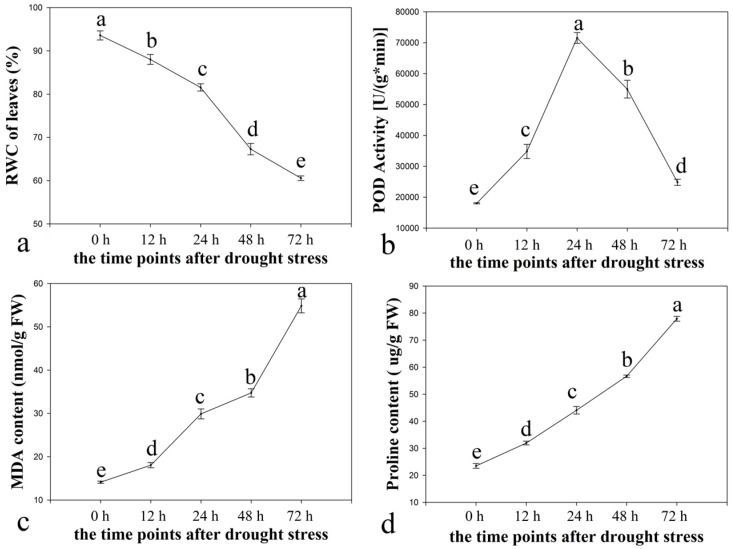
Four physiological traits of leaves from ramie seedlings under drought stress. (**a**) the RWC of leaves; (**b**) the POD activity; (**c**) the MDA content; (**d**) the proline content. The leaves were harvested at 0, 12, 24, 48, and 72 h after treatment with 15% (*w*/*v*) PEG6000. The error bars mean standard error. a, b, c, d, and e indicated significant differences (*p* < 0.05) among different time-points.

RWC seemed to decline consistently (Figure 1a), most significantly after 24 h. The highest RWC was at 0 h and the lowest RWC was at 72 h under drought stress. The critical time-points were observed as 0, 24, and 72 h after drought stress. The POD activity first increased but then decreased (Figure 1b). The highest POD activity was at 24 h and the lowest POD activity was at 0 h. The POD activity increased most significantly after 12 h and declined most significantly after 48 h. The POD activity remained stable after 72 h under drought stress. The critical time-point was 24 h after drought stress.

By contrast, the MDA and proline content increased throughout the entire drought stress periods (Figure 1c,d). The highest MDA and proline contents were at 72 h and the lowest were at 0 h under drought stress, the critical time-points were determined as 0 and 72 h after drought stress.

Based on the critical time-points of the change of the RWC, the POD activity, the MDA content, and the proline content under drought stress, we chose three time-points (0, 24, and 72 h after PEG treatment), to investigate the transcriptome.

### 2.2. Illumina Paired-End Sequencing, Reads Assembly, and Annotation

**Figure 2:**
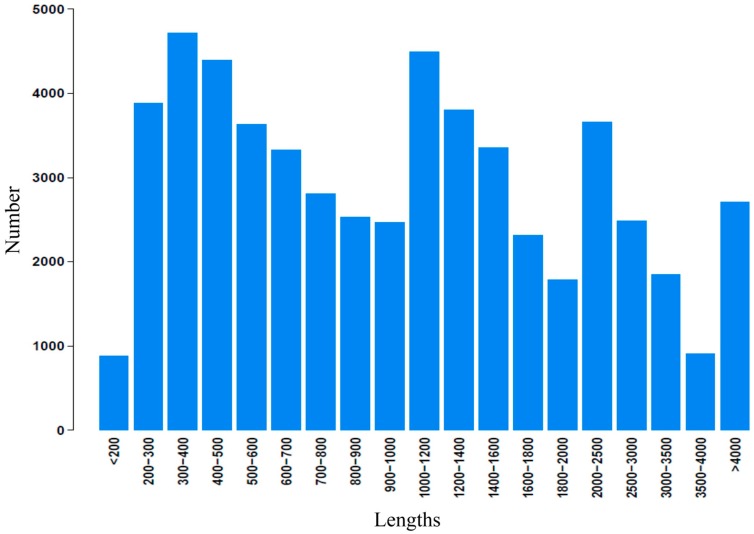
Length distribution of assembled contigs.

**Figure 3:**
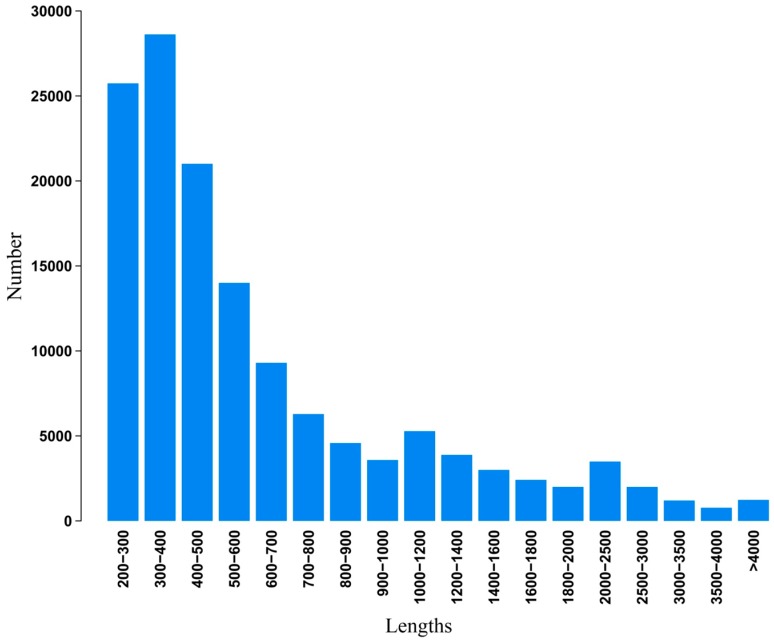
Length distribution of assembled unigenes.

Illumina paired-end sequencing technology yields 2 × 300 bp independent reads. After stringent quality checking and data cleaning, approximately 33,976,322,460 bp (30G) of high-quality data (94.02% of the raw data) were generated under the Q20 standard. The sequence data generated in this study have been deposited at the NCBI in the Short Read Archive database under accession SRP041143. Assembly of the high-quality sequencing reads yielded 138,381 unigenes, with an average length of 730.6 bp and a range from 201 to 20,860 bp. The lengths distribution of the assembled contigs is shown in Figure 2. The lengths distribution of the unigenes is given in Figure 3.

To provide putative annotations for the assembled unigenes, Blastp similarity searches were performed against the non-redundant protein (Nr) and the Swiss-Prot protein databases. The paired-end reads were realigned to contigs and the contigs in one transcript were assembled by Trinity and were defined as unigenes. The unigene sequences were compared to the non-redundant (nr) protein database with a cut-off E-value of 1 × 10^−5^. As a result, 47,565 (Supplementary file 1) unigenes (34%) were annotated. All sequences of the unigene are shown in Supplementary file 2.

### 2.3. Functional Classification and Metabolic Pathway Assignment

**Figure 4:**
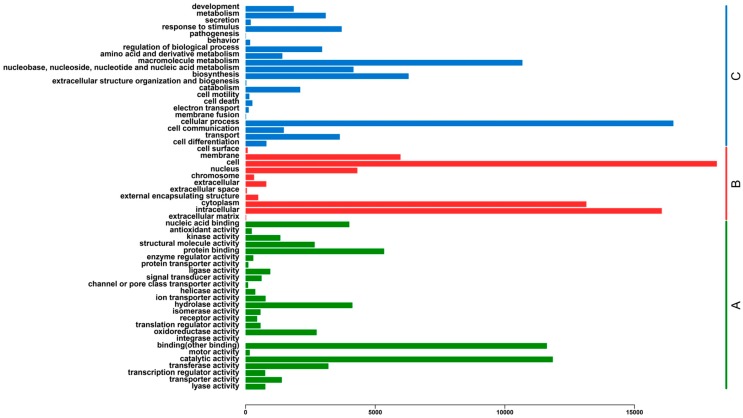
Histogram of Gene Ontology (GO) classifications. (**A**) Molecular function (green); (**B**) Cellular component (red); (**C**) Biological process (blue). The histogram indicates the number of unigenes that have GO annotations.

To provide putative functional classifications for the transcriptome assembly, all the assembled unigenes were evaluated using Blastp similarity searches against the Gene Ontology (GO) database, which classified 22,058 (Supplementary file 3) matched unigenes into the three functional categories (Figure 4). In molecular function, those that matched unique sequences were clustered into 25 subcategories. The largest subcategory in the molecular function class was “catalytic activity” (11,849; 21.54%) and the second was “binding” (11,620; 21.12%) (Figure 4). For the cellular component category, the sequences were divided into 11 subcategories. The most represented cellular components were “cell” (18,176; 30.58%) and “intracellular” (16,053; 27.01%) (Figure 4). For the biological process category, the sequences were classified into 21 subcategories. The most represented biological processes were “cellular process” (16,499; 27.67%) and “macromolecule metabolism” (10,676; 17.90%) (Figure 4).

**Figure 5:**
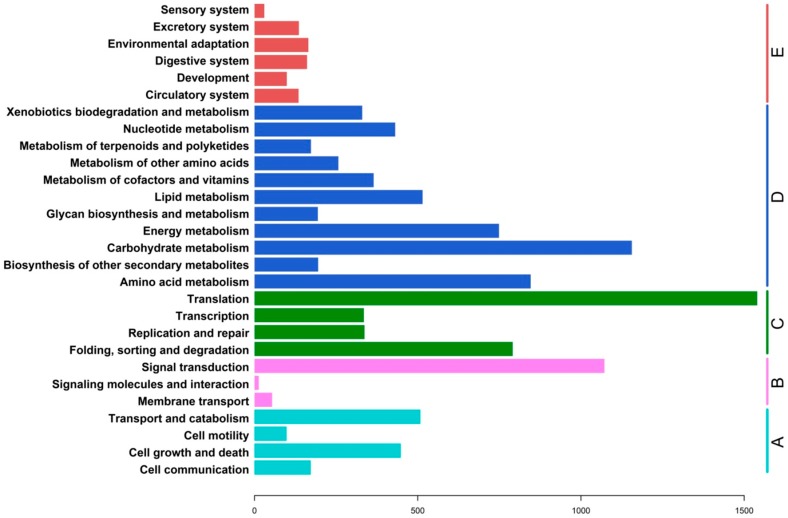
KEGG classification of assembled unigenes. A total of 6502 assembled sequences were associated with 2755 predicted KEGG metabolic pathways and were assigned to five KEGG biochemical pathways: (**A**) cellular processes; (**B**) environmental information processing; (**C**) genetic information processing; (**D**) metabolism; and (**E**) organismal systems.

The Kyoto Encyclopedia of Genes and Genomes (KEGG) [14] database can be used to analyze the gene products of metabolic processes. A total of 6502 (Supplementary file 4) assembled sequences were observed to be associated with 2755 predicted KEGG metabolic pathways, and were grouped into five KEGG biochemical pathways: Genetic information processing, organismal systems, cellular processes, environmental information processing and metabolism (Figure 5). The metabolic pathways were well represented by carbohydrate metabolism, amino acid metabolism, energy metabolism, lipid metabolism, nucleotide metabolism, metabolism of cofactors and vitamins, and the biodegradation and metabolism of xenobiotics. Those pathways related to genetic information processing included genes involved in translation, transcription, replication and repair, and folding, sorting and degradation. Pathways related to cellular processes and environmental information processing were also well represented by unigenes from ramie. These results represented a valuable resource for investigating metabolic pathways in ramie.

### 2.4. Analysis of Differential Gene Expression

Gene expression was calculated according to the reads per kilo base of transcript per million reads mapped (RPKM) method [15], using the MA-plot-based method with Random Sampling (MARS) model from the DEGseq [16] program package. 16,798 (Supplementary file 5) genes (12.14% of all genes) were identified as differentially expressed genes (DEGs) in roots and leaves. These amounted to 9842 genes (accounting for 7.11% of all genes) in the leaves of ramie and 9748 genes (accounting for 7.04%) in the roots. The numbers of unigenes that had differential expression patterns are shown in Table 1. Seven altered pathways were significantly enriched (corrected *p*-value ≤ 0.05), with genes involved in amino acid metabolism, carbohydrate metabolism, lipid metabolism, signal transduction, translation, energy metabolism, and folding, sorting, and degradation. These seven altered pathways were the most significantly enriched in the ramie leaves (Supplementary file 6); the *Y*-axis denotes the numbers of unigenes that were annotated to the enrichment between the two sample combinations in each KEGG pathway). For roots, the altered pathways contained xenobiotic biodegradation and metabolism genes, in addition to the seven altered pathways previously mentioned (Supplementary file 6).

**Table 1:** Numbers of unigenes that had differential expression patterns during drought stress. The comparisons were conducted from posterior samples to anterior ones. For example, the first row indicates that there were 2631 down-regulated unigenes and 1450 were up-regulated unigenes in sample L2 compared with sample L1.

Sample	Up-Regulated	Down-Regulated
L1–L2	1450	2631
L2–L3	2281	3120
R1–R2	3872	1913
R2–R3	2090	4590

### 2.5. Identification of Drought-Responsive TFs

**Figure 6:**
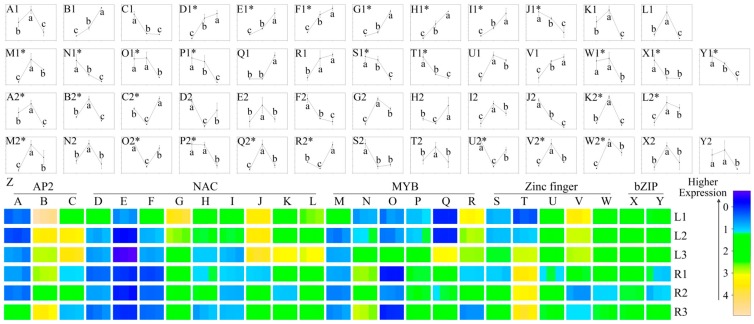
Expression analysis of 25 transcription factors. Specifically, comp79664, comp77101 and comp41385 (A, B and C, respectively) were from the AP2 TF family. Comp56509, comp79146, comp80892, comp72628, comp73235, comp77912, comp78350, comp75203 and comp80372 (D, E, F, G, H, I, J, K and L, respectively) were from the NAC TF family. Comp80737, comp86676, comp84340, comp51469, comp54867 and comp33749 (M, N, O, P, Q and R, respectively) were from the MYB TF family. Comp95985, comp83977, comp56037, comp83361 and comp52860 (S, T, U, V and W, respectively) were from the zinc finger TF family. Comp28477 and comp58004 (X and Y, respectively) were from the bZIP TF family. All of them were chosen for qRT-PCR quantification, using *GAPDH* as an internal control (Supplementary file 8). The line charts A1–Y1 were carried out in leaves, while A2–Y2 were carried out in roots. Each line chart (A1–Y2) was formed from the order of samples subjected to minor, moderate, and severe drought stress (*X*-axis) for their quantitative results (*Y*-axis) in three biological replicates. The significance analyses were performed for each unigene among three samples using the ANOVA method of Sigmaplot software with a cut-off *p*-value of 0.05. * indicate that the corresponding unigenes shared regular expression patterns from leaves (all-up or all-down) or roots (up–down or down–up) where they were picked out (Supplementary file 7). The error bars indicate the standard error.

Given that TFs appear to have a major effect on drought-responsive genes, one of the objectives of this study was to identify drought-inducible TFs. Among the differentially expressed unigenes, those with expression patterns that coincided with the patterns of the physiological traits (as displayed in Figure 1) were considered important. Generally, more unigenes were generated as “all-up” (unigenes up-regulated in L2 compared with L1, and simultaneously up-regulated in L3 compared with L2) or “all-down” patterns than “up-down” (unigenes were up-regulated in L2 compared with L1, while down-regulated in L3 compared with L2) and “down-up” patterns in leaves. While more TFs showed up-down or down-up patterns than all-up and all-down patterns in roots (Table 2). Accordingly, unigenes from the five main TF families (AP2, MYB, NAC, zinc finger, and bZIP) that shared all-up or all-down patterns in leaves and up-down or down-up patterns in roots were considered to have consistent physiological trait trends and were selected for further quantification. Altogether, 25 genes were found to encode known or putative TFs. Of the 25 TFs selected, three belonged to the AP2, six to the MYB, nine to the NAC, five to the zinc finger, and two to the bZIP families (Supplementary file 7). To further evaluate the role of these TFs, we analyzed their expression levels using quantitative real-time reverse transcription PCR (qRT-PCR). As shown in Figure 6, these TFs were all-up-regulated, all-down-regulated, up–down-regulated, or down–up-regulated by drought stress. Furthermore, when the transcriptome sequencing was performed, only one cDNA library was constructed for each sample; therefore, the effectiveness of the transcriptome data was validated (due to lack of duplication when preparing the transcriptome sequencing). Specifically, 11 unigenes (comp41385, C2; comp79146, E1; comp77912, I1; comp75203, K2; comp80737, M2; comp86676, N1; comp84340, O2; comp83977, T1; comp56037, U2; comp83361, V2 and comp58004, Y1 in Figure 6) from the 25 TFs were randomly chosen, and their expression profiles in leaves or roots (where they were picked out originally, as listed in Supplementary file 7) were tested (Supplementary file 8) using similarly treated plant samples. The results showed that they had the same expression trends (Supplementary file 9) with the qRT-PCR output from the residual RNA (Figure 6), and simultaneously correlated with the transcriptome data (Supplementary file 7). These data validated our transcriptome result.

**Table 2:** Numbers of unigenes with different expression patterns in leaves and roots during drought stress.

Samples	All-up	All-down	Up–down	Down–up
Leaves	379	637	348	98
Roots	28	57	3048	675

## 3. Discussion

### 3.1. Characterization of the Ramie Transcriptome

The Illumina sequencing method, with the largest output and lowest reagent cost, has been widely used for deep sequencing of model and non-model organisms [12,17,18]. In the previous studies, the Illumina paired-end sequencing platform was used for high-throughput sequencing of the ramie transcriptome [12,13]. There were some evident advantages to this study. First, after stringent quality checking and data cleaning, approximately 33,976,322,460 bp (30G) of high-quality data (94.02% of the raw data) were generated under the Q20 standard, which was more than previous studies in ramie [12,13]. Second, based on the high-quality reads, the sequencing assembly yielded 138,381 (Supplementary file 1) unigenes, which were more than that created by other transcriptome sequences. Third, 16,798 (Supplementary file 5) genes (12.14% of all genes) were identified as DEGs in roots or leaves, which comprised 9842 genes (accounting for 7.11% of all genes) in the leaves and 9748 genes (accounting for 7.04%) in the roots of ramie. The number of DEGs was higher than that found in previous studies [12,13]. Finally, the six samples, specifically the leaves (L1–L3) and roots (R1–R3) under increasing drought stress, were determined by the physiological traits (RWC *etc.*, as displayed in Figure 1) and phenotypes (data not shown). Thus, understanding on molecular regulation of ramie under drought stress was specifically ascertained by the results conducted from the ramie seedlings submitted to mild (L2 and R2) and severe (L3 and R3) drought stress compared with the control (L1 and R1). The transcriptome sequence generated in this study will be valuable for further ramie research on drought stress.

### 3.2. Physiological Traits Changed under PEG Treatment

The PEG-simulated drought approach has many advantages: The water potential (ψ_W_) can be controlled precisely and a large number of treatments can be performed quickly [19]. In this study, 15% (*w*/*v*) PEG6000 was used to induce drought stress. Many abiotic stresses trigger the production of ROS, which disrupt normal metabolism by oxidative damage of membrane lipids, proteins, and nucleic acids [20,21]. SOD and POD (among other so-called scavengers) are able to eliminate these harmful molecules [22]. In this study, more unigenes were generated as all-up or all-down patterns than up–down and down–up patterns in leaves. However, more up–down or down–up patterns than all-up and all-down patterns were observed for the root genes (Table 2), which coincided with the trends of the physiological traits (RWC, MDA, and proline content shared all-up or all-down trends, while POD activity was decreased after increasing, as indicated in Figure 1).

### 3.3. The Five Main Families of TFs Responding to Drought Stress in Ramie

Proteins that are characterized as being involved in the protection of plant cells from dehydration stress damage include molecule chaperons, osmotic adjustment proteins [23], ion channels [24], transporters [25] and antioxidation or detoxification proteins [26]. Transcriptome analyses using microarray technology, together with conventional approaches, have revealed that dozens of TFs are involved in the plant response to drought stress [27,28,29]. The expressions of these stress-related functional proteins are largely regulated by specific TFs [30,31]. More than 30 families of TFs have been predicted to be associated with drought stress from *Arabidopsis* [23]. Members of the NAC, AP2, MYB, bZIP, and zinc-finger families have been shown to have roles in the regulation of plant defense and stress responses [23,24,25,26,27].

Unigenes that shared all-up or all-down patterns in leaves and up-down or down-up patterns in roots were chosen for further quantitative analyses. Specifically, 25 TFs from the AP2 (3), MYB (6), NAC (9), zinc finger (5) and bZIP (2) families were putatively identified as directly associated with drought stress. Further expression analysis of these genes (Figure 6) and validation of randomly chosen unigenes (Supplementary file 9) by qRT-PCR confirmed their involvement in ramie’s response to drought stress and were consistent with the transcriptome data.

NAM (no apical meristem), ATAF1-2, and CUC2 (cup-shaped cotyledon) [32] constitute one of the largest TF families (NAC), and this family is plant-specific. NAC proteins play vital roles in many plant developmental processes [33] and also in biotic and abiotic stress tolerance [34,35]. The involvement of NAC TFs in the regulation of drought responses was first reported in *Arabidopsis* [35]; and NAC TFs have subsequently been shown to improve drought tolerance in rice [36] and cotton [7]. NAC TFs (encoding comp79664, comp77101, and comp41385) showed up–down or down–up expression patterns only in roots. Overexpression of the rice NAC gene *SNAC1* improved drought and salt tolerance by enhancing root development [7]. The proline content was enhanced. The MDA content was decreased in the transgenic cotton seedlings under drought and salt treatments compared with the wild-type [7]. In this study, the up–down or down–up expression of three genes (comp79664, comp77101, and comp41385) might be related with the increase of MDA and proline contents throughout the entire drought stress periods. In a previous report, 18 NAC domain factors were identified from expression profiling of stress-treated rice [37]. *OsNAC10* and *SNAC1/OsNAC9* were believed to have similar stress response functions because of their sequence similarity [37]. *OsNAC10*-overexpression in rice resulted in enlarged roots, and enhanced drought tolerance and grain yield under field drought conditions [37]. These results suggested the NAC TFs might confer drought resistance through altered root architecture.

The MYB TFs were previously reported to be regulatory genes in drought stress. They altered the expression levels of some drought stress-responsive genes and affected several physiological traits to overcome adverse conditions [38]. For example, *AtMYB2* plays a role in ABA-dependent drought stress responses [39]. The expression of *AtMYB41* is up-regulated when under drought, ABA or salt stress [40,41]. In a previous study, *AtMYB60* (an R2R3-MYB gene of *Arabidopsis*) was shown to be involved in the regulation of stomatal movements [42]. *AtMYB60* is a transcriptional modulator of physiological responses in guard cells, and its expression is negatively modulated during drought [42]. The water loss rate was lower in detached leaves from transgenic *Arabidopsis* compared with the WT leaves [43]. Overexpression of *AtMYB44* enhanced stomatal closure to confer abiotic stress tolerance in transgenic *Arabidopsis* [44]. *Arabidopsis, AtMYB88* and *AtMYB124* are also involved in the regulation of stoma aperture development [45,46]. In this study, a MYB TF (encoding comp80737) was up-regulated, while comp86676, comp84340, and comp51469 were down-regulated in leaves under drought stress. Comp54867 was first up-regulated and then down-regulated, whereas comp33749 was first down-regulated and then up-regulated in ramie roots under drought stress (Supplementary file 5). MYB TFs have different expression patterns in the leaves and roots of ramie. One MYB gene (*TaMYBsdu1*) was markedly up-regulated in the leaf and root of wheat under long-term drought stress [47]. These data suggested that MYB TFs might have different expression patterns in different crops. The MYB TFs might play an important role in the regulation of stomatal movements and improving water retention. This hypothesis will require further experimental testing.

The zinc finger TFs were previously reported to be positive regulators of plant tolerance to drought stress [48,49]. The transgenic lines that showed *DgZFP3*-overexpression (a drought stress-responsive Cys2/His2-type zinc finger protein gene) in tobacco plants did not accumulate as much H_2_O_2_ under drought stress. They accumulated more proline and showed greater POD and superoxide dismutase activities than in the WT plants under both the control and drought stress conditions [50]. DST (drought and salt tolerance) is a zinc finger transcription factor that negatively regulates stomatal closure via direct modulation of genes related to H_2_O_2_ homeostasis [51]. In this study, two zinc finger TFs (comp95985 and comp83977) were down-regulated in leaves under progressive drought stress (Supplementary file 5). They may be positive regulators in the increased accumulation of proline. Otherwise, two of them (comp83361 and comp52860) were first up-regulated and then down-regulated, whereas one (comp56037) was first down-regulated and then up-regulated in ramie roots under progressive drought stress (Supplementary file 5). We propose that the zinc finger TFs may play a key role in the increased accumulation of proline (Figure 1d) and in the improved peroxidase (POD) (Figure 1b) activities, which would enhance ramie tolerance to drought stress.

bZIP TFs were previously reported to be negative regulators of the drought stress response in rice [52] and tobacco [53], probably by deploying a better ROS-scavenging system to be an integral part of the defense against drought in the transgenic plants expressing *PtrABF* (a bZIP transcription factor) [53]. Our results (Figure 6X1,Y1) were consistent with previously reported results [52,53]. We inferred that the bZIP TFs might play an important role in reducing the accumulation of ROS and increasing the activities and expression levels of antioxidant enzymes (POD, Figure 1b) during drought stress.

The AP2/ERF family is a large family of plant-specific transcription factors that share a well-conserved DNA-binding domain [54]. This transcription factor family includes DRE-binding proteins (DREBs), which activate the expression of abiotic stress-responsive genes via specific binding of the dehydration-responsive element/C-repeat (DRE/CRT) cis-acting element in their promoters [54]. Transgenic alfalfa plants overexpressing *WXP1* (a putative *Medicago truncatula* AP2 domain-containing TF gene) showed improved drought tolerance compared with the wild-type [55]. Transgenic *Arabidopsis* plants indicated an AP2/EREBF TF (*AtERF7*) reduced the sensitivity of guard cells to ABA and increased transpirational water loss [56]. In this study, two AP2 TFs (comp79664 and comp77101) were first up-regulated and then down-regulated, whereas comp41385 was first down-regulated and then up-regulated in roots under progressive drought stress (Supplementary file 5). We hypothesize that the AP2 TFs might have important roles in ABA responses during drought stress. However, more evidence is needed to support this conclusion. The putative drought stress-related TFs that were identified in this study will further increase our understanding of gene expression, transcriptional regulation, and signal transduction during plant responses to drought. Our data will also help future investigations into drought adaptation in ramie. We have also developed an efficient regeneration and transformation protocol for ramie via *Agrobacterium*-mediated genetic transformation [57]. Furthermore, ongoing research in our laboratory using genetic engineering and several functional or regulatory genes has demonstrated activation or repression of both specific and broad pathways related to drought tolerance in ramie.

## 4. Material and Methods

### 4.1. Plant Materials Preparation, RNA Extraction, and cDNA Library Construction

In this study, the elite ramie variety, “Huazhu No. 5”, was used as the plant material and was obtained from the Ramie Germplasm Resources Garden, located at Huazhong Agricultural University, Wuhan, China. Two weeks after planting, the “Huazhu No. 5” seedlings were propagated from stem cuttings, which were transplanted into half-strength Hoagland’s solution for 20 days [7]. The period of seedling was when the plant height reached about 10 cm [7]. Physiological traits (the RWC, POD, the MDA, and the proline content) of ramie leaves at 0, 12, 24, 48, and 72 h after being subjected to drought stress (15% PEG6000) were measured to evaluate the severity of the stress. Every experiment was repeated at least three times. All stress experiments were conducted in a greenhouse at 28 ± 2 °C/23 ± 2 °C (day/night) with a relative humidity of 50%–70% under a 16/8-h (light/dark) photoperiod and a photon flux density of 350 µmol·m^−2^·s^−1^. Based on the changing trends of these parameters, we chose three time points, which were 0, 24, and 72 h after being treated with PEG, to investigate the transcriptome.

Total RNA was isolated from leaves and roots harvested at 0 (L1 and R1), 24 (L2 and R2), and 72 (L3 and R3) h, using an RNAprep Pure Plant Kit (Tiangen Biotech, Beijing, China) with three replicates. The RNA integrity and quality were confirmed by gel electrophoresis and using a NanoDrop 2000 spectrophotometer (Thermo, Waltham, MA, USA). Twenty micrograms of RNA were separately pooled from the six samples for cDNA library preparation. The residual RNA was used for qRT-PCR.

After digestion by DNase I (Takara, Japan) at 37 °C for 30 min, poly(A) RNA was isolated from 30 μg of total RNA using Dynabeads^®^ Oligo (dT) 25 (Life, Lake Success, NY, USA). The volume of purified mRNA was adjusted to 100 μL before adding 100 μL Binding Buffer, incubating at 65 °C for 2 min and immediately cooling on ice. Pre-rinsed magnetic beads (100 μL) were then added and the samples were then mixed well for 5 min at RT, and placed in the magnetic shelf (Life, Lake Success, NY, USA) for 1–2 min; the supernatant was then discarded. The magnetic beads were washed twice using 200 μL of Washing Buffer B, resuspended by 15 μL of Tris-HCl (10 mM, pre-cooled), and then heated at 75–80 °C for 2 min. The supernatant containing the purified mRNA was then generated using the magnetic shelf. The cDNA library was constructed from 100 ng of mRNA using NEBNext^®^ UltraTM RNA Library Prep Kit from Illumina (NEB, Ipswich, MA, USA), following the manufacturer’s instructions. The ligated cDNAs, ranging from 200 to 500 bp, were subjected to PCR amplification for 12–15 rounds using the Universal PCR Primer and the Index (X) Primer under the following procedures: 98 °C for 15 s, 65 °C for 30 s, and 72 °C for 30 s. Finally, the library was subjected to high-throughput sequencing after purification using an isometric volume of AMPure XP Beads (Agencourt, Webster, TX, USA).

### 4.2. Sequence Assembly, Annotation, and GO Terms/KEGG Pathways Construction

Transcriptome sequencing was performed for six equally pooled cDNA libraries, which were separately conducted from leaves (L1–L3) and roots (R1–R3) subjected to progressive drought stresses. Sequencing was performed on an Illumina HiSeq2500 genome analyzer. The raw reads were cleaned using FASTX-Toolkit (http://hannonlab.cshl.edu/fastx_toolkit/) to remove the adaptor sequences, empty reads and low-quality sequences (reads with unknown “N” sequences or those that were less than 20 bp long). The clean reads were assembled into non-redundant transcripts using Trinity [58]. The resulting sequences were used for Blastp searches and annotation against the Nr protein, the GO, the KEGG and the Swiss-Prot databases using an E-value cut-off of 1 × 10^−5^. The predicted protein coding sequences were annotated to the non-redundant protein databases in GenBank, Swiss-Prot, and TrEMBL using Blastp. GO mapping was carried out by GoPipe [59]. The KEGG pathway annotations were performed using Blast against the KEGG [14] databases.

### 4.3. Differential Expression Redundancy and Enrichment Analyses

After assembly and annotation, the universal reads from the six separately pooled samples (L1–L3, and R1–R3) were mapped to unigenes to calculate the RPKM value [15] for each assembled unigene. The differences in expression redundancy among the three samples from the leaves (L1–L3) or the roots (R1–R3) were determined using the RPKM value [15] for each unigene using the MARS model from the DEGseq program package [16]. The *p* value threshold was <0.001. The differentially expressed genes were used for GO terms/KEGG pathway enrichment analyses using the hyper geometric test to measure significantly enriched terms:

(1) $$P=1-\overset{m-1}{\underset{i=0}{\sum }}\frac{\left(\begin{array}{c} M\\ i \end{array}\right)\left(\begin{array}{c} N-M\\ n-i \end{array}\right)}{\left(\begin{array}{c} N\\ n \end{array}\right)}$$

where *N* is the number of genes with GO/KEGG annotations and *n* represents the number of differentially expressed genes in *N*. The variables, *M* and *m*, represent the total number of genes and the number of differentially expressed genes, respectively, in each GO/KEGG term. The threshold used to determine significant enrichment of the gene sets was corrected to a *p* value ≤0.05 and a false discovery rate (FDR) <0.01.

### 4.4. Real-Time Quantitative RT-PCR

The residual RNA from transcriptome sequencing was used for qRT-PCR. The RNA was reverse transcribed into cDNA using the GoScript Reverse Transcription System (Promega, Fitchburg, WI, USA), following the manufacturer’s protocol. QRT-PCR was performed in a 20-μL reaction vessel containing 10 μL iTaq Universal SYBR Green supermix (Bio-RAD; Hercules, CA, USA), 10 pmoL of forward and reverse gene-specific primers, and 1 μL cDNA. Forward and reverse primers (Supplementary file 8) were designed for each gene of interest using Primer 3 online software version 4.0.0 [60] and adjusted by oligo software version 7.56 [61]. Additionally, primers specific to *GAPDH* were designed to serve as an endogenous control to normalize the data for differences in input RNA. PCR amplification was performed in an IQ5 (Bio-Rad, Hercules, CA, USA), according to the manufacturer’s instructions. Following a denaturation step at 95 °C for 5 min, the amplification step comprised 40 cycles at 95 °C for 15 s and 60 °C for 30 s. A melting curve was constructed to determine the specificity of each PCR primer by maintaining the reaction at 95 °C for 1 min, cooling the sample to 55 °C for 1 min and finally heating to 95 °C at a rate of 0.5 °C per 6 s. The reactions were carried out in triplicate to ensure reproducibility.

### 4.5. Estimations of the RWC, the MDA, the Proline Content, and POD

The RWC was calculated according to the following method [48]:

RWC (%) = (FW − DW)/(TW − DW) × 100
(2)

FW, fresh leaf weights; TW, turgid leaf weights; DW, dry leaf weights.

The MDA levels (a measure of lipid peroxidation) were assessed as previously described [7] by measuring the amount of thiobarbituric acid reactive substances present in the samples. MDA content (μmoL·L^−1^) was calculated using the formula:

C (μmoL·L^−1^) = 6.45 × [(OD_532_ − OD_600_)] − 0.56 × OD_450_ (3)

The extraction and colorimetric determination of proline from leaves were carried out according to a previously described method [39].

POD was determined using the guaiacol oxidation method as previously described [48].

## 5. Conclusions

In this study, we used Illumina paired-end sequencing technology to characterize the transcriptome of ramie under progressive drought stress. After stringent quality checking and data cleaning, approximately 33,976,322,460 bp (30G) of high-quality data (94.02% of raw data) were generated from 170 million raw sequence reads. Differential gene expression analysis revealed 9281 putative genes in leaves and 8627 in roots that might be associated with drought tolerance. Among these, 25 TFs shared consistent expression patterns with physiological traits associated with drought stress, and thus they were picked out and validated by qRT-PCR. This study represents a fully characterized transcriptome, and provides a valuable resource for genetic and genomic studies in ramie plants, especially under drought stress. Our work is useful for breeding drought-resistant ramie varieties.

## Supplementary Materials

Supplementary materials can be found at http://www.mdpi.com/1422-0067/16/02/3493/s1.
